# The lymphocyte/monocyte ratio predicts the efficacy of isatuximab plus pomalidomide in multiple myeloma patients

**DOI:** 10.1007/s00262-024-03711-8

**Published:** 2024-05-17

**Authors:** Yutaka Shimazu, Junya Kanda, Yoshiyuki Onda, Shin-ichi Fuchida, Kensuke Ohta, Yuji Shimura, Satoru Kosugi, Ryosuke Yamamura, Mitsuhiro Matsuda, Hitoshi Hanamoto, Yoko Adachi, Naoyuki Anzai, Masaaki Hotta, Kentaro Fukushima, Hideo Yagi, Satoshi Yoshihara, Yasuhiro Tanaka, Teruhito Takakuwa, Hirokazu Tanaka, Hirohiko Shibayama, Nobuhiko Uoshima, Naoki Hosen, Tomoki Ito, Chihiro Shimazaki, Itaru Matsumura, Junya Kuroda, Akifumi Takaori-Kondo, Masayuki Hino

**Affiliations:** 1 Department of Hematology and Oncology Graduate School of Medicine, Kyoto University, 54, Kyoto, Kawaramachi, Shogoin, Sakyoku 606-8507 Japan; 2 Kyoto Innovation Center for Next Generation Clinical Trials and iPS Cell Therapy, Kyoto University Hospital, Kyoto, Japan; 3 Department of Early Clinical Development, Graduate School of Medicine, Kyoto University, Kyoto, Japan; 4 Department of Hematology, Osaka Red Cross Hospital, Osaka, Japan; 5 Department of Hematology, Japan Community Health Care Organization Kyoto Kuramaguchi Medical Center, Kyoto, Japan; 6 Hematology Ohta Clinic, Shinsaibashi, Japan; 7 Division of Hematology and Oncology, Department of Medicine, Kyoto Prefectural University of Medicine, Kyoto, Japan; 8 Department of Hematology, Toyonaka Municipal Hospital, Toyonaka, Japan; 9 Department of Hematology, Osaka Saiseikai Nakatsu Hospital, Osaka, Japan; 10 Department of Hematology, PL General Hospital, Osaka, Japan; 11 Department of Hematology, Kindai University Nara Hospital, Ikoma, Japan; 12 Department of Internal Medicine, Japan Community Health Care Organization Kobe Central Hospital, Kyoto, Japan; 13 Department of Hematology, Uji Tokushukai Hospital, Uji, Japan; 14 First Department of Internal Medicine, Kansai Medical University, Hirakata, Japan; 15 Department of Hematology and Oncology, Osaka University Graduate School of Medicine, Osaka, Japan; 16 Department of Hematology and Oncology, Nara Prefecture General Medical Center, Nara, Japan; 17 Division of Hematology, Department of Internal Medicine, Hyogo College of Medicine, Hyogo, Japan; 18 Department of Hematology, Shinko Hospital, Kobe, Japan; 19 Department of Hematology, Osaka Metropolitan University Graduate School of Medicine, Osaka, Japan; 20 Department of Hematology and Rheumatology, Faculty of Medicine, Kindai University, Higashiosaka, Japan; 21 Department of Hematology, National Hospital Organization Osaka National Hospital, Osaka, Japan; 22 Department of Hematology, Japanese Red Cross Kyoto Daini Hospital, Kyoto, Japan

**Keywords:** Multiple myeloma, Isatuximab, Lymphocyte/monocyte ratio, Predictive markers

## Abstract

**Background:**

Isatuximab, an anti-CD38 antibody, has been widely used in treatments for patients with relapsed/refractory multiple myeloma (MM). Despite its high efficacy, not all patients achieve a lasting therapeutic response with isatuximab.

**Objective:**

We tried to identify biomarkers to predict the effectiveness of isatuximab by focusing on the host's immune status before treatment.

**Methods:**

We retrospectively analyzed the cases of 134 relapsed/refractory MM patients in the Kansai Myeloma Forum database who had received only a first isatuximab treatment.

**Results:**

Among the 134 patients, an isatuximab, pomalidomide and dexamethasone (Isa-PD) regimen, isatuximab, carfilzomib and dexamethasone (Isa-KD) regimen and isatuximab and/or dexamethasone (Isa-D) regimen were used in 112, 15 and 7 patients, respectively. The median age at treatment, number of prior treatment regimens, and progression-free survival (PFS) were 71, 6, and 6.54 months, respectively. Multivariate analysis showed that the PFS under the Isa-PD regimen was longer in patients with higher lymphocyte/monocyte ratio (LMR ≥ 4), fewer prior treatment regimens (< 6), and no use of prior daratumumab treatment. The OS under the Isa-PD regimen was longer in patients with higher white blood cell counts (WBC counts ≥ 3000/μL) and higher LMR. The PFS under the Isa-D regimen was longer in patients with fewer prior treatment regimens in univariate analysis, but no parameters were correlated with PFS/OS under the Isa-KD regimen.

**Conclusion:**

We found that the patients with higher LMR (≥ 4) could obtain longer PFS and OS under the Isa-PD regimen. Other cohort studies of isatuximab treatment might be necessary to substantiate our results.

**Supplementary Information:**

The online version contains supplementary material available at 10.1007/s00262-024-03711-8.

## Introduction

The prognosis of multiple myeloma (MM) patients has been dramatically improved by proteasome inhibitors, immunomodulatory drugs, and anti-CD38 antibodies [1–5]. Among them, isatuximab, one of the new anti-CD38 antibodies, has shown a high response rate with a superior prognosis for relapsed/refractory MM patients when used in combination with pomalidomide, carfilzomib and dexamethasone [3–5]. However, although 60% to 80% of MM patients responded to isatuximab treatment in clinical trials [3–5], some patients did not benefit sufficiently from isatuximab treatment, and a certain number of patients could not obtain a therapeutic response. Although immunotherapies are increasingly playing a role in MM treatment [6], we do not have any appropriate specific biomarkers that could predict the response or the durable efficacy of immunotherapies such as isatuximab before treatment [7].

To identify patients who might benefit from isatuximab before treatment, we focused on the immunological aspect of isatuximab. The mechanisms of action of isatuximab are immune-mediated effects, such as complement- or antibody-dependent cell-mediated cytotoxic effects, antibody-dependent cell phagocytic activity, depletion of CD38-positive regulatory immune cells, and direct killing activity of antibodies against myeloma cells [8–11]. Previously, we reported that pretreatment lymphocyte or monocyte counts could predict the efficacy of other antibodies used for the treatment of MM, such as elotuzumab and daratumumab [12, 13], or bispecific T-cell engager antibody [14]. Also, several studies have indicated that the lymphocyte/monocyte ratio (LMR) could predict the prognosis of MM patients [15, 16]. Here, we hypothesized that the immune conditions (as represented by white blood cell counts LMR, and neutrophil/lymphocyte ratio) before isatuximab treatment might predict its efficacy. As a proof of concept, we conducted a retrospective observational analysis using real-world data from the Kansai Myeloma Forum (KMF) database in Japan.

## Methods

### Study design and participants

KMF, a study group consisting of 131 physicians in 43 facilities in Japan, established a database that includes physician-reviewed, real-world clinical data on the diagnosis, treatment, and periodical follow-up of patients with plasma cell dyscrasias. This study was approved by the Kyoto University Graduate School and Faculty of Medicine Ethics Committee (approval no. R2887). A total of 4,814 patients with plasma cell dyscrasias were registered in the KMF database in September 2023. All these patients were diagnosed as having MM or MM-related disorders based on institutional assessment. For purposes of the present retrospective analysis, we selected the patients who were older than 20 years, had relapsed MM, and had been treated with an isatuximab-containing regimen between August 2020 and August 2023 (after its June 2020 approval for clinical use in Japan). A total of 112 patients administered a total of 128 isatuximab treatments met the above criteria (Supplementary Fig. 1). We selected the patients who had received only a first administration of isatuximab and omitted the 14 patients with a second and 2 patients with a third isatuximab treatment. We thus analyzed 112 relapsed MM patients who had undergone only first isatuximab treatment and were followed until October 2023.

The patients' responses to treatment were assessed based on the international uniform response criteria [17] for multiple myeloma. The patients' best responses against isatuximab were classified by institutional physicians into five categories: complete response (CR), very good partial response (VGPR), partial response (PR), stable disease (SD), and progressive disease (PD). For the high-risk cytogenetic abnormalities, we adopted the abnormalities reported in the International Myeloma Working group consensus statement [18], such as deletion 17p, t(4;14) and t(14;16). Unfavorable cytogenetic abnormalities were categorized by a fluorescence in situ hybridization (FISH) analysis. CD138 purification was not performed for FISH analysis, and patients with 20% positive cells were considered positive for FISH analysis.

### Statistical methods

We calculated the progression-free survival (PFS) for isatuximab treatment as the time from isatuximab treatment until the date of progression of MM, death by any cause or the date of last contact as a primary endpoint. The data were censored for the date of last administration of isatuximab in cases with planned isatuximab cessation. The laboratory data 1–7 days before cycle 1 day 1 isatuximab treatment after the previous treatment were used. We determined the cutoff values using the 25th, 50th and 75th percentile values (Supplementary Fig. 2A-E and 3) with reference to the previous studies [12–16]. We set the secondary endpoint as overall survival (OS).

The survival curves based on the PFS and OS curve were plotted using the Kaplan–Meier method, and the log-rank test was used for comparisons among groups. The Cox proportional hazard model was used to calculate the hazard ratio for each variable along with the 95% confidence interval (CI). All the variables were applied in the univariate analysis and a multivariate analysis was conducted for the variables which showed a p value of less than 0.1 in the univariate analysis. We used the bootstrap method [19, 20] to validate our multivariate analysis results for the variables that showed a p value of less than 0.1 in the univariate analysis **(**Table 3). In each step, 1000 bootstrap samples with replacements were created from the dataset [19, 20]. All statistical analyses were performed using the EZR (ver. 1.61) software package (Saitama Medical Center/Jichi Medical University, Saitama, Japan) [21] along with a graphical user interface for the R software package (ver. 4.2.2; The R Foundation for Statistical Computing) or SPSS software ver. 29.02 (IBM, USA). P-values < 0.05 were considered significant in all analyses.

## Results

### Progression-free survival of isatuximab in relapsed multiple myeloma

The characteristics of the patients undergoing each regimen are summarized in Table 1 and Supplementary Table 1. In brief, a total of 134 patients who were treated with isatuximab for the first time were analyzed. The median age at the time of isatuximab treatment was 71 (range: 34–87) years old. The numbers of patients treated with the Isa-PD, Isa-KD and Isa-D regimens were 112 (83.6%), 15 (11.2%) and 7 (5.2%), respectively. The median number of prior regimens was 6 and daratumumab had been used before isatuximab treatment in 84 cases (63.6%): 69 cases with an Isa-PD regimen, 13 cases with an Isa-KD regimen and 2 cases with an Isa-D regimen. The histograms of laboratory data before isatuximab treatment are shown in Supplementary Fig. 2. Patients with a CR, VGPR or PR were regarded as having a therapeutic response to isatuximab; these included 44 patients (39.3%) treated with the Isa-PD regimen, 5 cases (33.3%) treated with the Isa-KD regimen and 1 case (16.7%) treated with the Isa-D regimen (Supplementary Fig. 3).

**Table 1:** Patient characteristics for the whole cohort

Type of treatment regimen		
	Isa-PD	112 (85.6%)
	Isa-KD	15 (11.2%)
	Isa-D	7 (5.2%)
Age (years) at treatment		
	Median (range)	71 (34–87)
Gender		
	Male	71 (54.2%)
	Female	60 (45.8%)
Type of heavy chain		
	IgG	81 (60.9%)
	IgA	25 (18.8%)
	BJP	21 (15.8%)
	IgM	1 (0.8%)
	IgD	3 (2.3%)
	NA	2 (1.5%)
Type of light chain		
	λ	87 (64.9%)
	κ	44 (32.8%)
	NA	3 (2.2%)
ISS stage at diagnosis		
	I	42 (31.3%)
	II	42 (31.3%)
	III	39 (21.6%)
	NA	21 (15.7%)
High-risk cytogenic abnormality		
	None	58 (43.3%)
	At least one	47 (35.1%)
	NA	29 (21.6%)
Laboratory data before isatuximab treatment		
White blood cell count	(/μL, median, range)	4580 (1240–13230)
Lymphocyte/monocyte ratio	(median, range)	2.36 (0.05–193.0)
Neutrophil/lymphocyte ratio	(median, range)	2.30 (0.03–67.0)
Free light chain	(mg/L, median, range)	
	κ	34.3 (0.5–12,040)
	λ	10.1 (0.4–4020)
	κ/λ ratio	3.1 (0.001–4089)
B2MG	(mg/L, median, range)	2.7 (0.17–14.34)
Prior regimen numbers		
	Median (range)	6 (2–18)
Prior use of daratumumab		
	Yes	84 (63.6%)
Follow-up period of survivor		
	Median days (range)	472 (7–1137)

The characteristics of multiple myeloma patients treated with isatuximab regimens are shown in Table 1. Laboratory data were collected before the isatuximab treatment

*NA* not available, *ISS* International Staging system; β_2_ microglobulin: B2MG

**Fig. 1:**
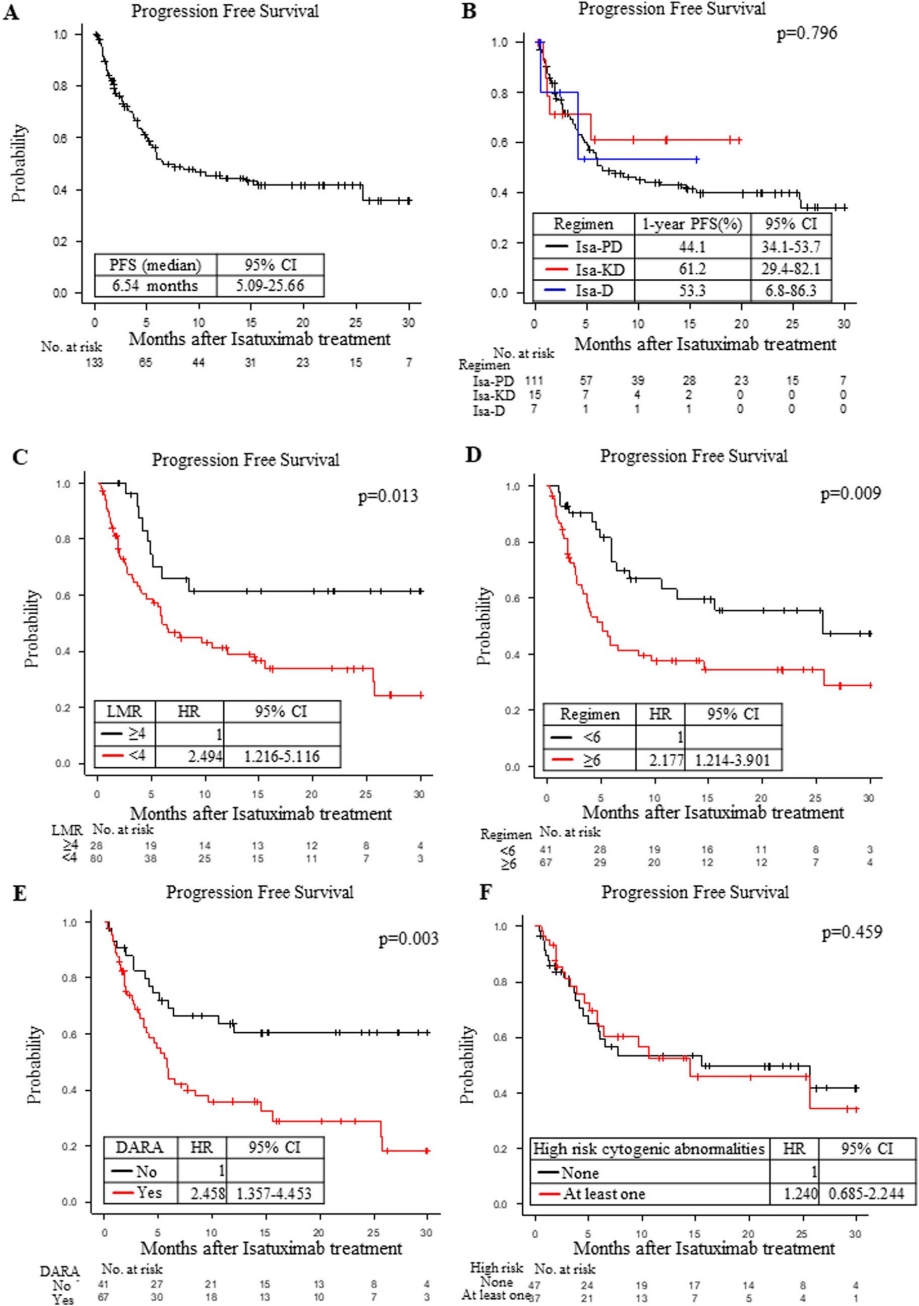
**A** The progression-free survival (PFS) of the multiple myeloma (MM) patients treated with isatuximab. The entire cohort was used to calculate PFS. Median PFS (months) values with the 95% CI (confidence interval) are shown. **B** The PFS of the MM patients under the following regimens: isatuximab, pomalidomide and dexamethasone (Isa-PD, *black*); isatuximab, carfilzomib and dexamethasone (Isa-KD, *red*); and isatuximab and dexamethasone (Isa-D, *blue*). One-year PFS values (%) with the 95% CI are shown. **C** The PFS of the MM patients under the Isa-PD regimen according to the lymphocyte/monocyte ratio (LMR): 4 or more (*black*) or less than 4 (*red*). The hazard ratio (HR) with the 95% CI is shown. The survival curves were adjusted by the significant factors in the multivariate analysis. **D** The PFS of the MM patients under the Isa-PD regimen according to the number of previous regimens: 6 or more (*black*) or less than 6 (*red*). The hazard ratio (HR) with the 95% CI is shown. The survival curves were adjusted by the significant factors in the multivariate analysis. **E** The PFS of the MM patients under the Isa-PD regimen according to the prior use of daratumumab (DARA): No (*black*) or Yes (*red*). The hazard ratio (HR) with the 95% CI is shown. The survival curves were adjusted by the significant factors in the multivariate analysis. **F** The PFS of the MM patients under the Isa-PD regimen according to the high risk cytogenic abnormalities: none (*black*) or at least one (*red*). The hazard ratio (HR) with the 95% CI is shown. The survival curves were adjusted by the significant factors in the multivariate analysis. The number of patients at risk in each group is shown in the lower panel of each figure

**Fig. 2:**
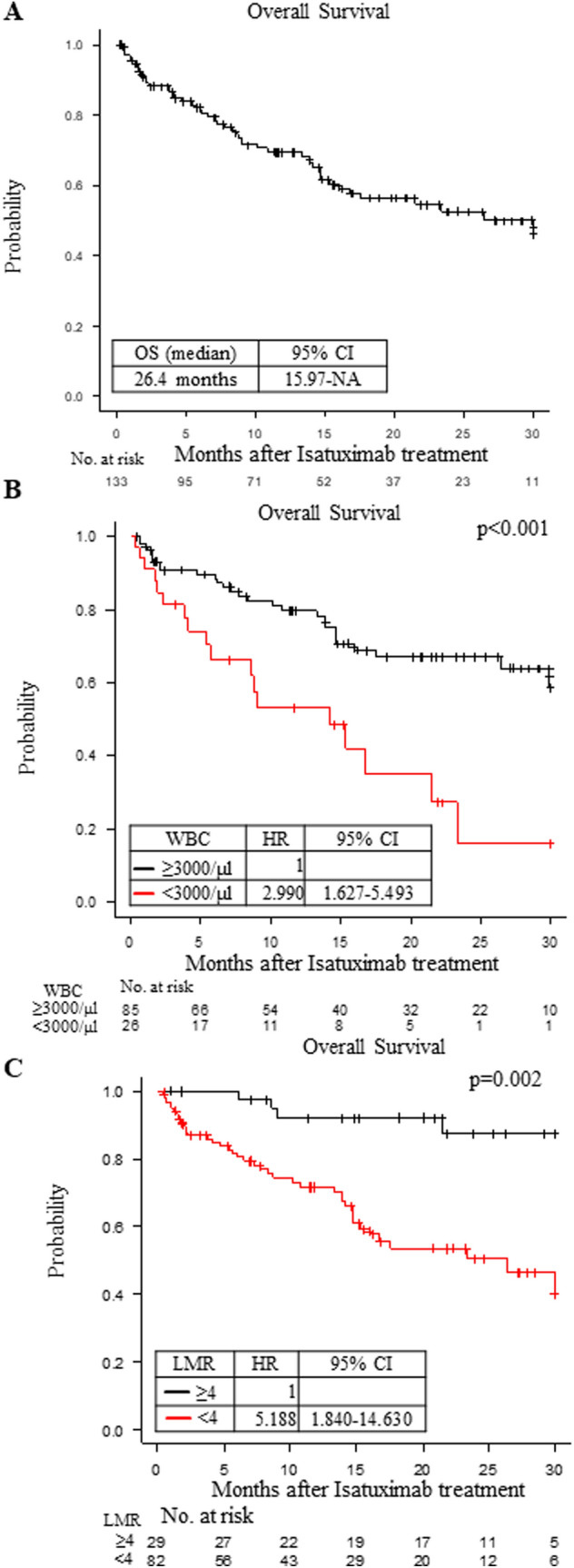
**A** The overall survival (OS) of the MM patients for the entire cohort. Median OS (months) with the 95% CI is shown. **B** The overall survival (OS) of the MM patients under the Isa-PD regimen according to the white blood cell (WBC) counts: 3000/μl or more (*black*) or less than 3000/μl (*red*). The hazard ratio (HR) with the 95% CI is shown. The survival curves were adjusted by the significant factors in the multivariate analysis. **C** The OS of the MM patients under the Isa-PD regimen according to the lymphocyte/monocyte ratio (LMR): 4 or more (*black*) or less than 4 (*red*). The hazard ratio (HR) with the 95% CI is shown. The survival curves were adjusted by the significant factors in the multivariate analysis. The number of patients at risk in each group is shown in the lower panel of each figure

The median PFS under isatuximab treatment was 6.54 (95%CI: 5.09–25.66) months in this cohort (Fig. 1A). When we compared the PFS by the regimens, the 1-year PFS ratios under the Isa-PD, Isa-KD and Isa-D regimens were 44.1% (34.1–53.7), 61.2% (29.4–82.1) and 53.3% (6.8–86.3), respectively (Fig. 1B; not significant). The median OS under isatuximab treatment was 26.4 (95%CI: 15.97-Not available) months in this cohort (Fig. 2A).

### Underlying factors affecting the PFS and OS under the Isa-PD regimen

We analyzed the underlying factors affecting the PFS under each treatment regimen. We found that the PFS of patients under the Isa-PD regimen was longer in those with higher white blood cell (WBC) count (≥ 3000/μl; p = 0.048), higher LMR (≥ 4; p = 0.002), lower neutrophil/lymphocyte ratio (NLR < 2.3; p = 0.020), lower β_2_ microglobulin level (B2MG < 3.5 mg/L; p = 0.009), and lower prior regimen number (< 6; p = 0.013) and in those not using daratumumab prior to isatuximab treatment (p = 0.041; Table 2 and Fig. 1C-F). The PFS of patients under the Isa-D regimen was longer in those with a lower prior regimen number (< 6; p = 0.046, Supplementary Table 2), but we could not find any factors which correlated to PFS under the Isa-KD regimen (Supplementary Table 2).

**Table 2:** Univariate analysis for progression-free survival and overall survival under the Isa-PD regimen

Univariate analysis		PFS					OS				
Factors		1-year-PFS (%)	95% CI	Hazard ratio	95% CI	*P*-value	1-year-OS (%)	95% CI	Hazard ratio	95% CI	*P*-value
Age	< 65 years	41.4	22.8–59.1	1		0.906	76.4	56.8–88.0	1		0.434
	≥ 65 years	43.4	31.6–54.7	0.967	0.556–1.682		65.5	52.9–75.4	1.301	0.671–2.523	
Gender	Male	38.4	25.2–51.5	1		0.144	69.0	54.3–79.8	1		0.607
	Female	50.2	35.2–63.6	0.685	0.411–1.143		69.4	54.4–80.4	0.859	0.481–1.535	
High risk cytogenic abnormalities	None	51.7	35.4–65.8	1		0.280	76.2	60.1–86.5	1		0.674
	At least one	42.5	25.6–58.4	1.240	0.685–2.244		67.1	49.2–79.9	1.338	0.695–2.579	
	NA	33.3	15.9–51.9	1.667	0.884–3.144		60.0	36.8–77.0	1.232	0.564–2.693	
White blood cell counts	≥ 3000/μl	49.4	37.5–60.3	1		0.048	76.3	65.0–84.4	1		< 0.001
	< 3000/μl	27.9	12.1–46.2	1.732	0.999–3.011		47.7	27.3–65.6	2.824	1.551–5.143	
Lymphocyte/monocyte ratio	≥ 4	63.9	42.0–79.3	1		0.002	88.3	67.9–96.1	1		< 0.001
	< 4	37.1	26.1–48.2	2.960	1.455–6.023		62.3	50.1–72.3	5.523	1.975–15.440	
Neutrophile/lymphocyte ratio	< 2.3	54.6	39.9–67.1	1		0.020	78.7	64.8–87.6	1		0.011
	≥ 2.3	32.6	19.6–46.3	1.822	1.089–3.048		59.3	44.1–71.7	2.152	1.177–3.936	
ISS stage	I	42.8	25.9–58.7	1		0.935	69.3	50.2–82.2	1		0.832
	II	44.2	25.3–61.6	1.764	0.720–4.318		62.9	42.8–77.6	2.864	0.880–9.315	
	III	47.9	29.3–64.3	3.402	1.364–8.484		67.0	47.1–80.8	7.153	2.297–22.270	
κ/λ ratio	0.1–10	58.6	36.4–75.4	1		0.074	81.2	60.4–91.7	1		0.072
	≤ 0.1, ≥ 10	38.3	26.1–50.4	1.783	0.937–3.396		64.3	50.9–75.0	1.993	0.920–4.317	
B2MG	< 3.5 mg/L	59.4	38.7–75.2	1		0.009	82.1	62.3–92.1	1		0.007
	≥ 3.5 mg/L	39.4	22.1–56.4	2.636	1.270–5.471		60.4	40.8–75.3	3.113	1.347–7.190	
Prior regimen numbers	< 6	62.6	44.1–76.5	1		0.003	82.6	65.2–91.9	1		0.006
	≥ 6	33.9	22.5–45.6	2.326	1.310–4.129		60.5	47.3–71.4	2.581	1.280–5.203	
Prior use of daratumumab	No	62.2	44.5–75.7	1		0.003	78.7	61.8–88.8	1		0.026
	Yes	33.9	22.2–45.9	2.378	1.322–4.278		63.1	49.7–73.8	2.089	1.078–4.046	

Progression-free survival (PFS) was calculated from the time of isatuximab treatment to the progression of the disease. Overall survival (OS) was calculated from the time of isatuximab treatment to the time of death by any cause. Univariate analyses against PFS and OS under the Isa-PD regimen were performed for each factor. The log-rank test was used for comparisons among groups. One-year-PFS (%) with the 95% confidence interval (CI), hazard ratio with the 95% CI and *P*-value are shown

*PFS* progression-free survival, *OS* overall survival, *CI* confidence interval, *ISS* International Staging System, *B2MG* β_2_ microglobulin, *NA* not available

We next analyzed the underlying factors affecting the OS under each treatment regimen. We found that the OS of patients under the Isa-PD regimen was longer in those with higher white blood cell (WBC) counts (≥ 3000/μl, p < 0.001), higher LMR (≥ 4; p < 0.001), lower NLR (< 2.3; p = 0.011), lower B2MG(< 3.5 mg/L; p = 0.007), and lower prior regimen number (< 6; p = 0.006) and in those not using daratumumab prior to isatuximab treatment (p = 0.026; Table 2). We could not find any factors that were correlated with OS under the Isa-KD and Isa-D regimens (Supplementary Table 3).

### Higher LMR was correlated with both better PFS and better OS under the Isa-PD regimen

We performed a multivariate analysis of the PFS in patients undergoing the Isa-PD regimen by analyzing all factors that had p values less than 0.1 in the univariate analysis (Table 3). We found that higher LMR (≥ 4; p = 0.013), lower prior regimen number (< 6; p = 0.009) and not using daratumumab prior to isatuximab treatment (p = 0.003) were associated with significantly superior PFS under the Isa-PD regimen (Table 3), and these results were confirmed by the bootstrap method (Table 3). The PFS values of patients undergoing the Isa- PD regimen are shown according to LMR, prior regimen number and prior use of daratumumab in Fig. 1C, D and E.

**Table 3:** Multivariate analyses of PFS and OS under the Isa-PD regimen

Factors		PFS				OS			
		Hazard ratio	95% CI	*P*-value	*P*-value*	Hazard ratio	95% CI	*P*-value	*P*-value*
White blood cell counts	≥ 3000/μl	1				1			
	< 3000/μl	1.721	0.970–3.056	0.064	0.081	2.990	1.627–5.493	< 0.001	0.002
Lymphocyte/monocyte ratio	≥ 4	1				1			
	< 4	2.494	1.216–5.116	0.013	0.009	5.188	1.840–14.630	0.002	< 0.001
Neutrophil/lymphocyte ratio	< 2.3	1				1			
	≥ 2.3	1.409	0.790–3.907	0.231		1.467	0.782–2.751	0.231	
κ/λ ratio	0.1–10	1				1			
	≤ 0.1, ≥ 10	1.058	0.502–2.228	0.883		1.116	0.468–2.659	0.805	
	NA	1.181	0.442–3.159	0.740		0.916	0.271–3.100	0.887	
B2MG	< 3.5 mg/L	1				1			
	≥ 3.5 mg/L	1.927	0.849–4.375	0.117		1.512	0.601–3.809	0.380	
	NA	1.837	0.876–3.850	0.107		1.357	0.579–3.182	0.107	
Prior regimen numbers	< 6	1				1			
	≥ 6	2.177	1.214–3.901	0.009	0.012	1.838	0.897–3.768	0.097	0.065
Prior use of daratumumab	No	1				1			
	Yes	2.458	1.357–4.453	0.003	0.011	2.014	1.032–3.929	0.040	0.062

Multivariate analyses of PFS and OS under the Isa-PD regimen were performed using the factors that showed *P* < 0.1 in univariate analysis. The Cox proportional hazard model was used to calculate the hazard ratio for each variable; the 95% CI and p-value are shown. *The *P*-value after the bootstrapping process (1000 samples) using the factors that showed *P* < 0.1 in multivariate analysis

*PFS* progression-free survival, *OS* overall survival, *CI* confidence interval, *B2MG* β_2_ microglobulin

In multivariate analysis for the OS in patients undergoing the Isa-PD regimen, higher WBC counts (≥ 3000/μl; p < 0.001), higher LMR (≥ 4; p = 0.002) and not using daratumumab prior to isatuximab treatment (p = 0.040) were associated with significantly superior OS (Table 3). The results of higher WBC counts and higher LMR were also confirmed by the bootstrap method (Table 3). The OS in the full cohort of patients undergoing the Isa-PD regimen are shown according to WBC counts and LMR in Fig. 2B and C. The PFS or OS under Isa-KD and Isa-D did not change according to LMR (Supplementary Table 2–3 and Supplementary Fig A-B).

### The influence of prior use of daratumumab on treatment with the Isa-PD regimen

**Fig. 3:**
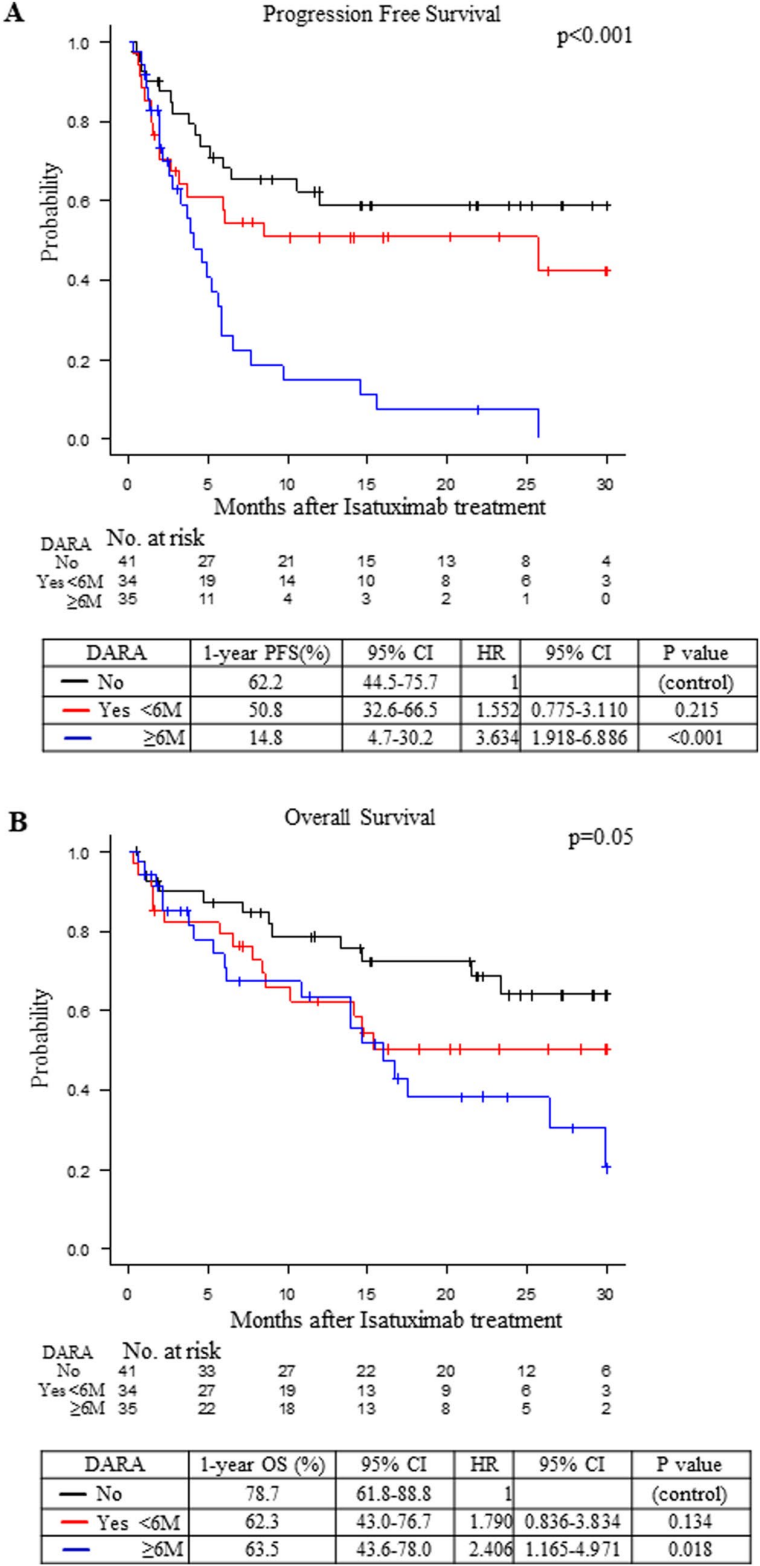
**A** The PFS of the MM patients under the Isa-PD regimen according to prior use of DARA: No (*black*); Yes but treated with isatuximab less than 6 months after the last DARA treatment (*red*); and Yes but treated with isatuximab 6 months or later after the last DARA treatment (*blue*). One-year PFS (%) with the 95% CI, the HR with 95% CI, and the p-value are shown. **B** The OS of the MM patients under the Isa-PD regimen according to prior use of DARA: No (*black*); Yes but treated with isatuximab less than 6 months after the last DARA treatment (*red*); and Yes but treated with isatuximab 6 months or later after the last DARA treatment (*blue*). One-year PFS (%) with the 95% CI, the HR with 95% CI, and the p-value are shown. The number of patients at risk in each group is shown in the lower panel of each figure

Because the prior use of daratumumab had a negative impact on prognosis after the Isa-PD regimen, we analyzed the interval between last daratumumab treatment and the Isa-PD regimen (≥ 6 months or < 6 months). The PFS and OS under the Isa-PD regimen were significantly shorter in patients who had received the Isa-PD regimen 6 months or more after the last daratumumab treatment than in the patients without prior use of daratumumab (p < 0.001 and p = 0.018, respectively; Fig. 3A-B). However, the PFS and OS under the Isa-PD regimen were not significantly different between patients for whom the Isa-PD regimen was used less than 6 months after the last daratumumab treatment and patients without prior use of daratumumab (not significant, Fig. 3A-B).

## Discussion

The effectiveness of immunotherapies against MM could be influenced by the immune status of the host, but there is a lack of useful biomarkers to predict the clinical response before treatment [6, 7]. We have previously reported that the efficacy of elotuzumab and daratumumab could be predicted by lymphocyte and monocyte counts, respectively [12, 13]. Also, several studies have indicated that LMR might predict the prognosis of MM patients [15, 16]. In this study, we demonstrated that LMR (≥ 4) easily predicted the longer PFS and OS of the Isa-PD regimen in relapsed/refractory MM patients. This result was consistent with previous reports [15, 16].

We also found that the patients with lower prior regimen number (< 6) and not using daratumumab prior to isatuximab treatment showed longer PFS under the Isa-Pd regimen. It is not surprising that the PFS under the Isa-PD regimen would be shorter in heavily treated patients with treatment-resistant MM, as fewer prior regimens has been associated with better prognosis in a clinical study [3–5]. We demonstrated that patients with fewer than 6 prior treatment regimens showed significantly longer PFS under the Isa-D regimen, but not under the Isa-KD regimen. This might be due to the limited sample size; we thus considered that the results for the Isa-KD and Isa-D regimens were only exploratory in nature. Also, we showed that the effectiveness of isatuximab was attenuated by the prior use of daratumumab treatment. It has been reported that the number of immune cells, such as NK cells, could be decreased by the use of daratumumab treatment [22–27]. The elimination of myeloma cells by isatuximab depends not only on a direct antibody effect but also on a complement- or antibody-dependent cell-mediated cytotoxic effect [8–11]. Therefore, the prior use of daratumumab might attenuate the effectiveness of isatuximab. As higher WBC counts were correlated with superior OS under the Isa-PD regimen, another explanation could be that higher LMR and WBC counts might be a prerequisite along with a higher number of immune cells; this should be confirmed in future studies. We also considered that the prior treatment to the isatuximab treatment might have influenced WBC counts or LMR. However, we could not find a correlation between the number of prior regimens and WBC counts or LMR (**Supplementary Fig. 6A-B**). The PFS under the Isa-PD regimen was shorter in patients who had received the Isa-PD regimen 6 months or more after their last daratumumab treatment than in patients who had received the Isa-PD regimen less than 6 months after their last daratumumab treatment (Fig. 3A). Because the choice of treatment depended on individual physicians and the OS was not significantly different between the two groups (Fig. 3B), we speculated that the remaining sensitivity against the anti-CD38 antibody might have differed between the two groups. Although the prior use of daratumumab had a negative impact on PFS under the Isa-PD regimen, simply extending the interval between the last daratumumab administration and the next isatuximab treatment did not appear to restore the efficacy of isatuximab.

Despite these results, we should underscore that isatuximab treatment remains a high priority treatment option for all MM patients due to its high response rate [3–5], even for the relapsed/refractory low LMR patients, who might have a suppressed immune status. Once patients relapse or become refractory to treatments, the effective duration of the next treatments could become shorter, and their prognosis would be much worse [28]. Utilizing the LMR before treatment with an Isa-PD regimen might provide two important pieces of information. First, patients with LMR < 4 have a suppressed immune status and might experience attenuated or unsustained efficacy of isatuximab. Second, the physicians of patients with a suppressed immune status (LMR < 4) might need to prepare for the next treatment after the Isa-PD regimen. Because this was an observational study, we could not tell whether we could change the prognosis of the patients by choosing a treatment other than the Isa-PD regimen. We speculate that LMR is not a prognostic marker in general but a biomarker for the Isa-PD regimen because LMR was difficult to apply for either the Isa-KD or Isa-D regimen in this study. However, this might have been due to the small sample size of patients on the Isa-KD and Isa-D regimen, which needs to be verified by other datasets.

There were several limitations in this study. First, this was a retrospective observational study, and the individual physicians made all the treatment choices. Thus, there may have been a bias toward the choice of isatuximab treatment that could not be captured in the multivariate analysis. Second, because of the limited number of analyzed patients, we could not divide the patients into a derivation cohort and validation cohort for analysis. We adopted a bootstrap method for the internal validation, but it was difficult to confirm the external validation in our cohort. Therefore, we need to substantiate our results by other datasets. Third, we could not examine the effect of prior treatment on LMR and other laboratory data. Fourth, we could not analyze the detailed fractions of white blood cells, lymphocytes, and monocytes (such as CD4 + T cells, CD8 + T cells, regulatory T cell, natural killer cells, etc.) for a deeper understanding of the mechanism of isatuximab. Despite these limitations, we found that the patients with higher LMR (≥ 4) could obtain longer PFS and OS under the Isa-PD regimen. Other cohort studies of isatuximab treatment might be necessary to substantiate our results.

### Supplementary Information

Below is the link to the electronic supplementary material.

Supplementary file1 (PDF 562 kb)

## Data Availability

The data of this study are available from the corresponding author, J. Kanda, upon reasonable request.

## Abbreviations

B2MG: β_2_ Microglobulin
CI: Confidence interval
CR: Complete response
Isa-D: Isatuximab and dexamethasone
Isa-KD: Isatuximab, carfilzomib and dexamethasone
Isa-PD: Isatuximab, pomalidomide and dexamethasone
ISS: International Staging system
KMF: Kansai Myeloma Forum
LMR: Lymphocyte/monocyte ratio
MM: Multiple myeloma
NA: Not available
NLR: Neutrophile/lymphocyte ratio
OS: Overall survival
PD: Progressive disease
PFS: Progression-free survival
PR: Partial response
SD: Stable disease
VGPR: Very good partial response
WBC: White blood cell
