# Supplementary material for: The lymphocyte/monocyte ratio predicts the efficacy of isatuximab plus pomalidomide in multiple myeloma patients
Source: Cancer Immunol Immunother. 2024 May 17;73(7):135. doi: 10.1007/s00262-024-03711-8 (PMC11101389; doi:10.1007/s00262-024-03711-8)
Supplement: Supplementary file 1 — Supplementary file1 (PDF 562 kb) [file 262_2024_3711_MOESM1_ESM.pdf]

Supplemental Fig 1.

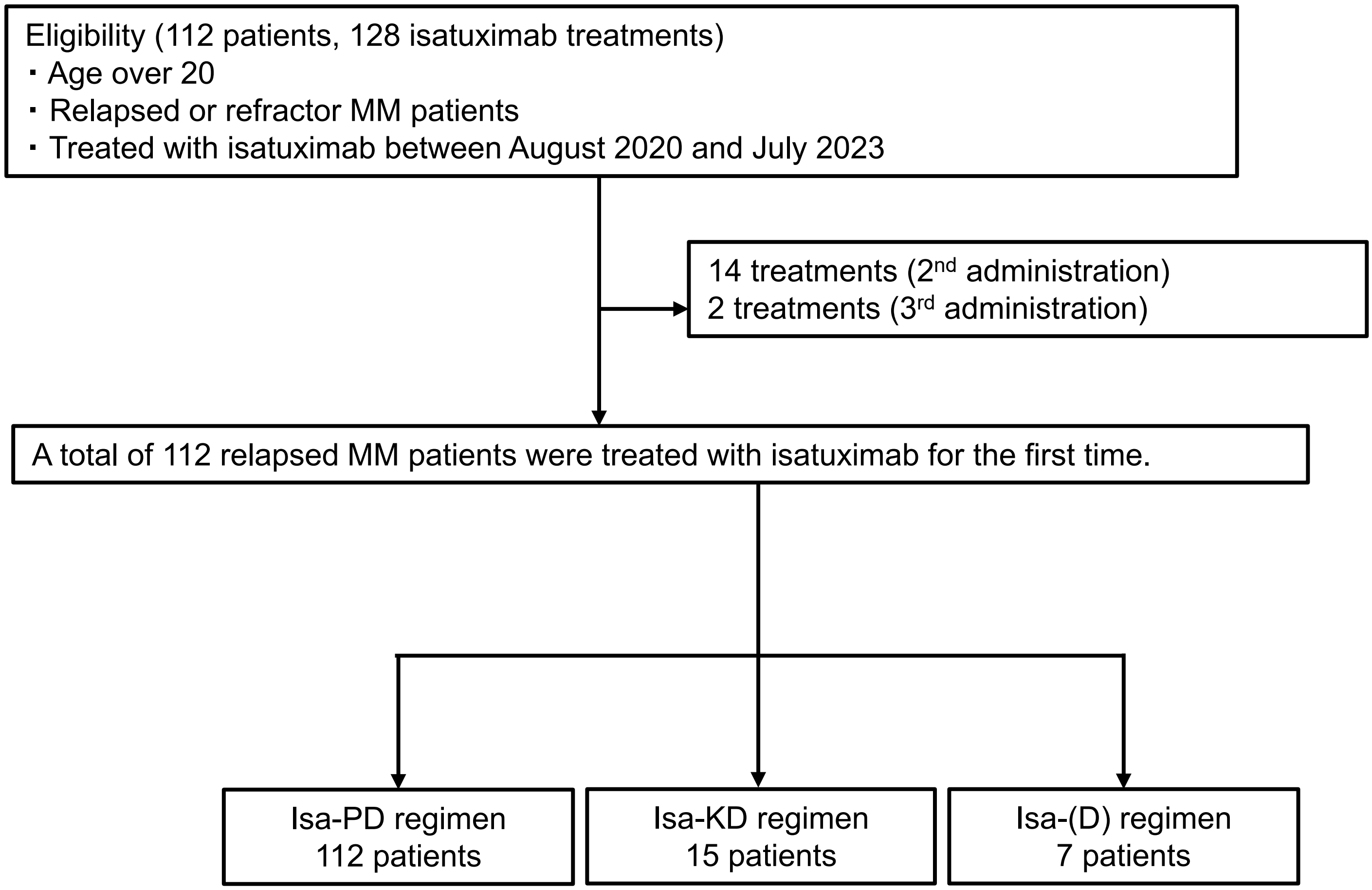

Supplemental Fig 1. Consort diagram of the study.

Supplemental Fig 2.

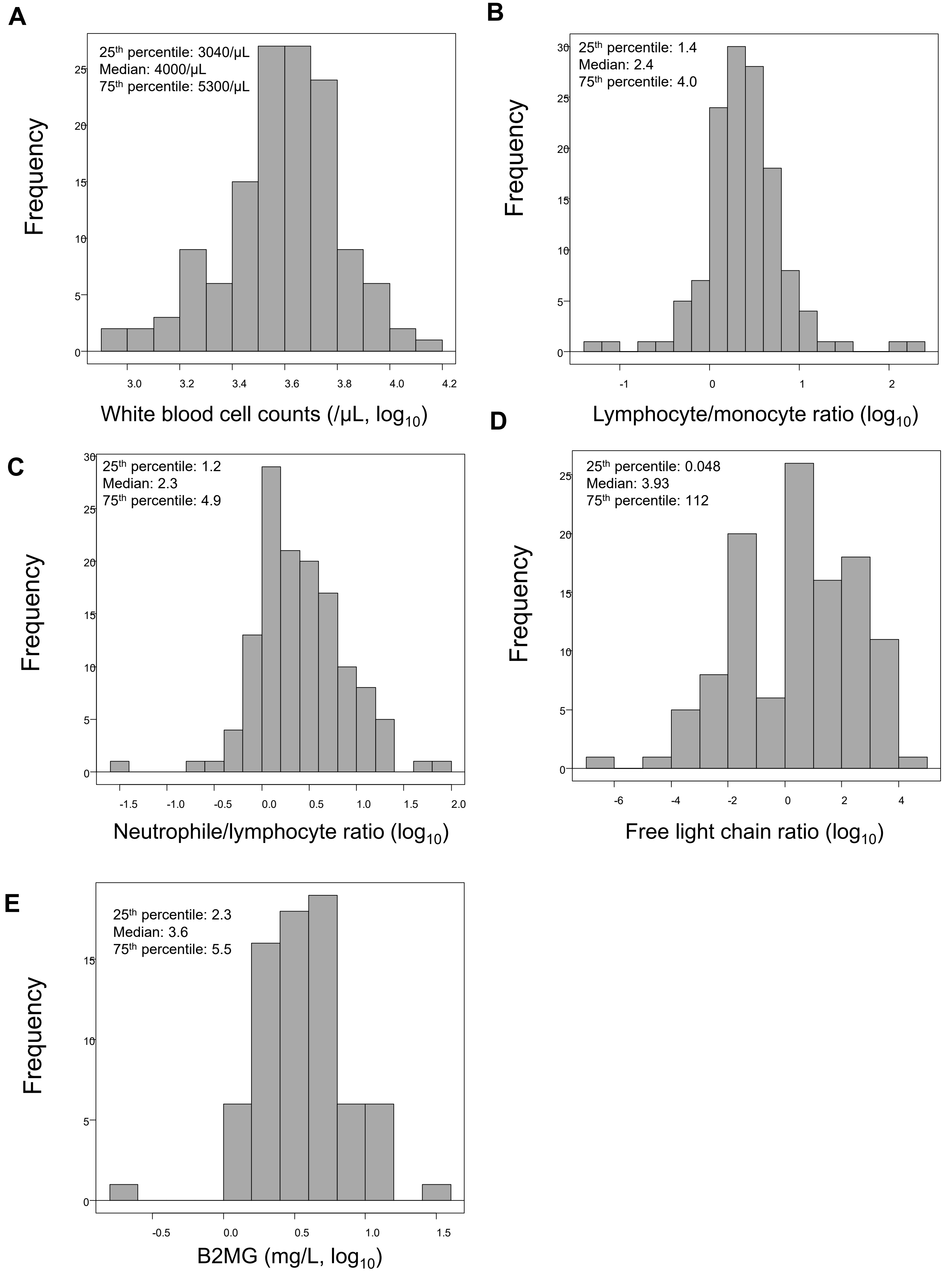

Supplemental Fig 2. (A-F) Histograms of the white blood cell counts **(A)**, lymphocyte/monocyte ratio **(B)**, neutrophil/lymphocyte ratio **(C)**, free light chain ratio **(D),** and β_2 microglobulin (B2MG) **(E)**. The horizontal axis is plotted in log scale. The 25th percentile, median, and 75th percentile values are described in the upper left panel of each figure.

Supplemental Fig 3.

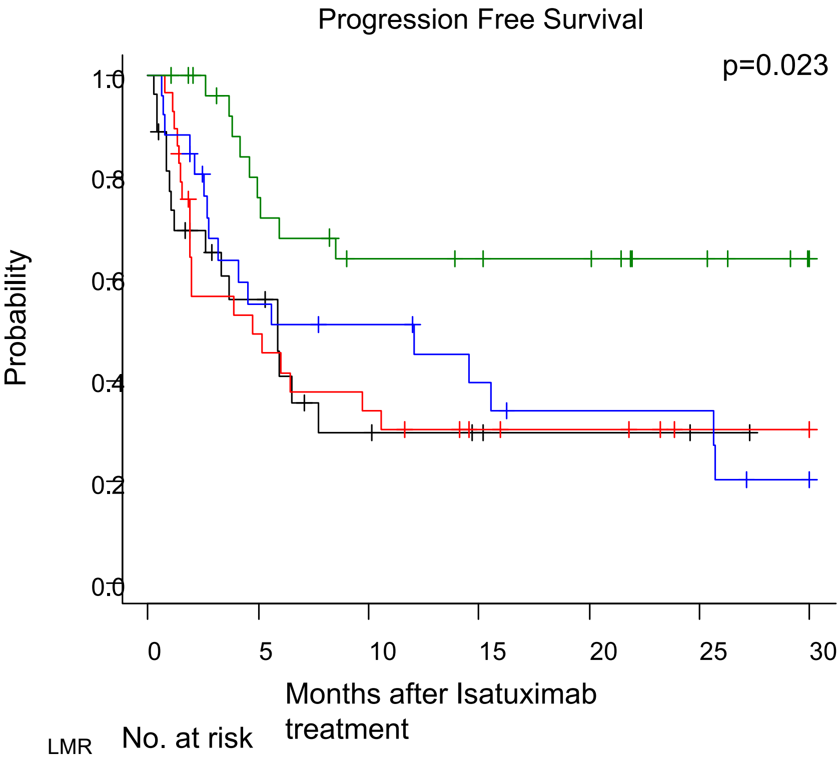

LMR	No. at risk						
<1.4	27	12	5	3	2	1	0
1.4-2.4	29	13	9	5	4	1	1
2.4-4.0	26	13	11	7	5	5	2
≥4.0	29	19	14	13	12	8	4

LMR	HR	95% CI	P value
— <1.4	1		(control)
— 1.4-2.4	0.959	0.492-1.868	0.902
— 2.4-4.0	0.800	0.402-1.591	0.525
— ≥4.0	0.307	0.135-0.699	0.005

Supplemental Fig 3. The progression-free survival (PFS) of the multiple myeloma (MM) patients under an isatuximab, pomalidomide, and dexamethasone (Isa-PD) regimen according to the lymphocyte/monocyte ratio (LMR): <1.4 (*black*), 1.4–2.4 (*red*), 2.4–4.0 (*blue*), and ≥4.0 (*green)*. The hazard ratio (HR) with the 95% confidence interval (CI) and p-value are shown. The number of patients at risk in each group is shown in the lower panel of each figure.

Supplemental Fig 4

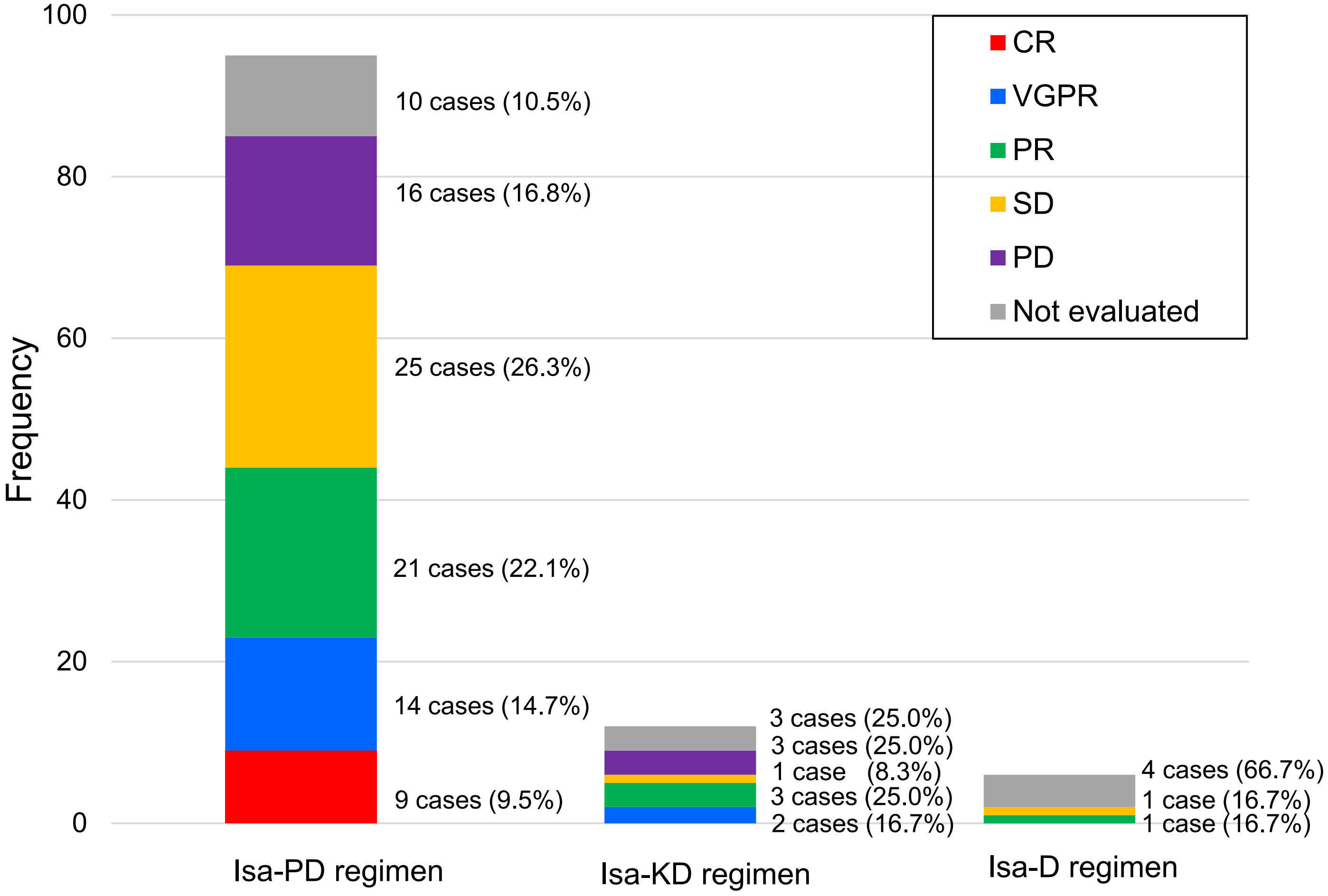

Supplemental Fig 4. The proportion of the best treatment response against isatuximab treatment. The number of cases (with %) of each regimen is shown. Patients with a CR, VGPR or PR were regarded as having a therapeutic response to isatuximab.

Abbreviations: CR, VGPR, PR, SD, PD, Isa-PD, Isa-KD and Isa-D. CR: complete remission; VGPR: very good partial response; PR: partial response; SD: stable disease; PD: progressive disease; Isa-PD: isatuximab, pomalidomide, and dexamethasone; Isa-KD: isatuximab, carfilzomib, and dexamethasone; Isa-D: isatuximab and dexamethasone.

Supplemental Fig 5.

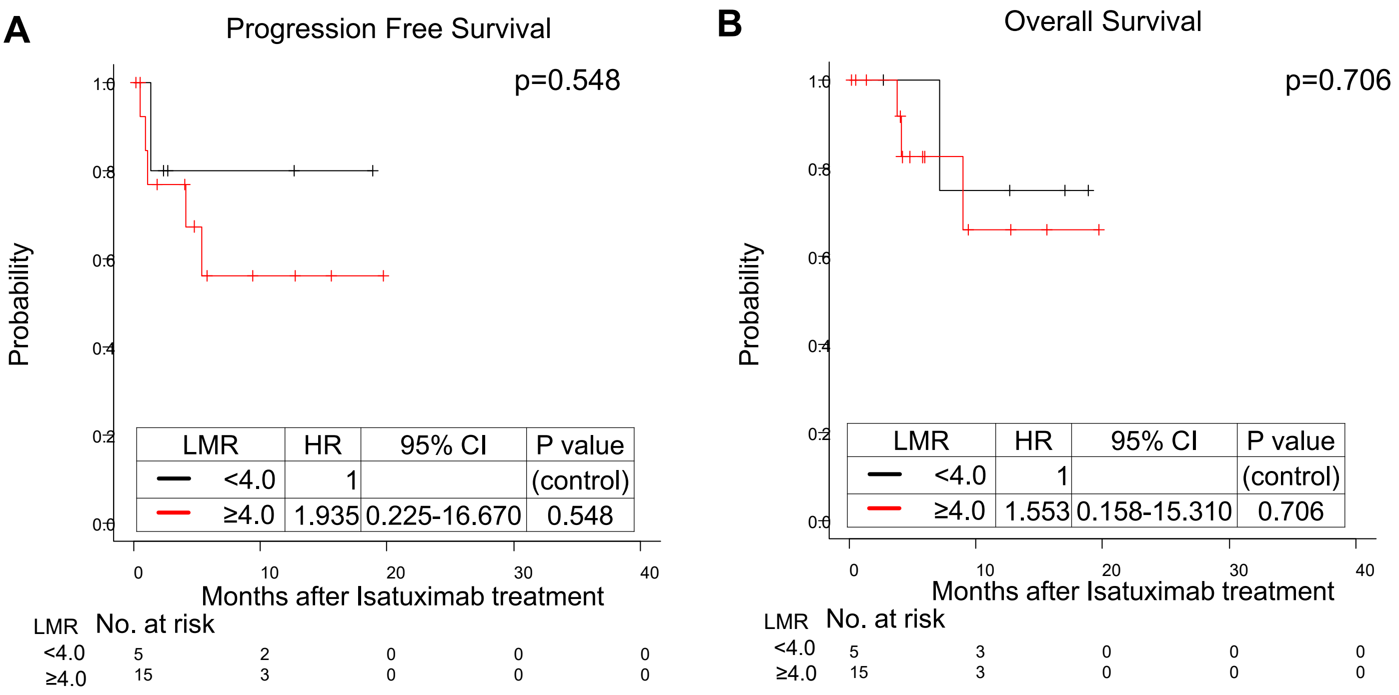

Supplemental Fig 5(A-B). The PFS (**A**) or OS (**B**) of the MM patients under an isatuximab, carfilzomib, and dexamethasone (Isa-KD) and an isatuximab and dexamethasone (Isa-D) regimen according to the lymphocyte/monocyte ratio (LMR): <4.0 (*black*) and ≥4.0 (*red)*. The hazard ratio (HR) with the 95% confidence interval (CI) and p-value are shown. The number of patients at risk in each group is shown in the lower panel of each figure.

Supplemental Fig 6.

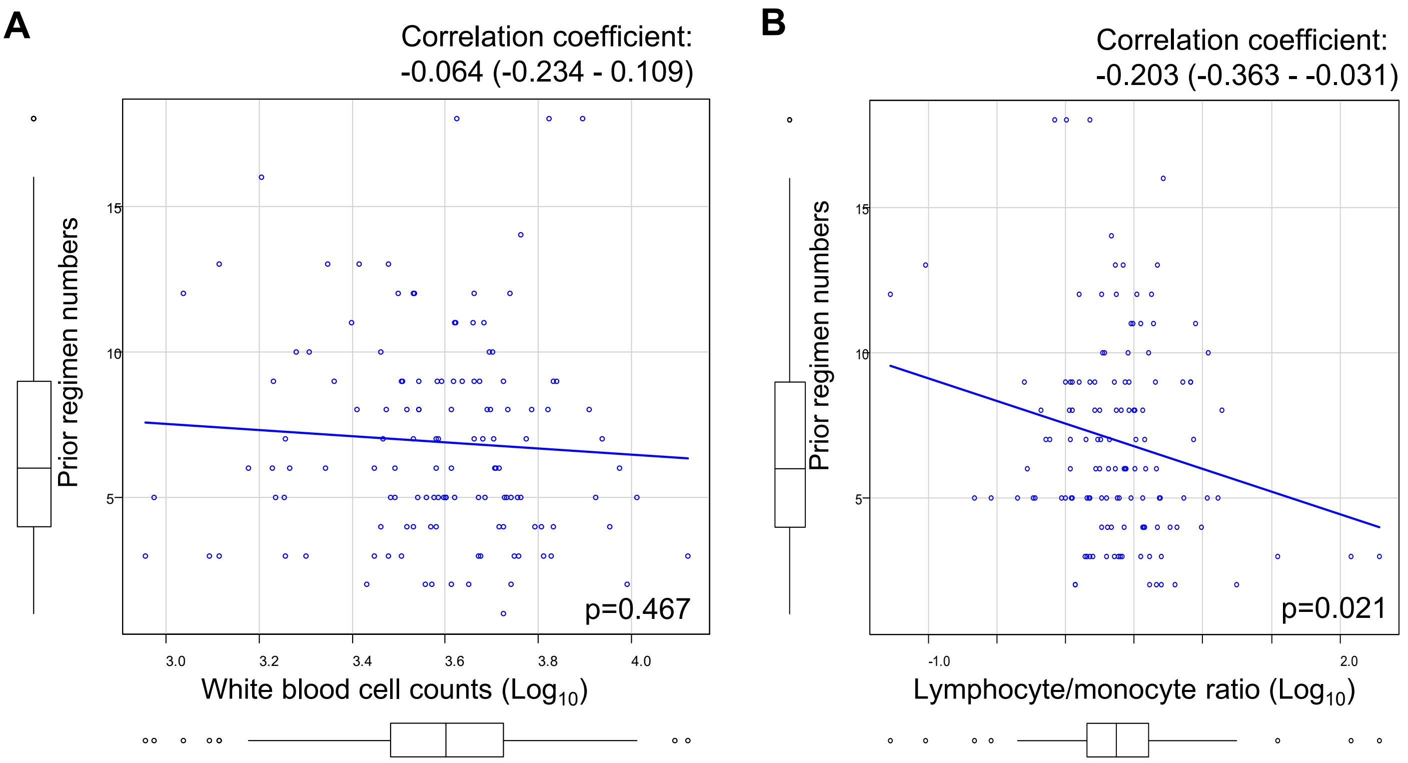

Supplemental Fig 6(A-B). The correlation between prior regimen numbers and white blood cell counts **(A)** or lymphocyte/monocyte ratio **(B)** was analyzed by Person's correlation coefficient.

Supplementary Table 1. Patient characteristics according to each regimen

Type of treatment regimen		Isa-PD	Isa-KD	Isa-D	p-value
Number of patients					
		112	15	7	
Age (years) at treatment					0.916
	median (range)	71 (42-89)	71 (34-83)	72 (50-81)	
Gender					0.525
	male	58 (51.8%)	9 (60.0%)	5 (71.4%)	
	female	54 (48.2%)	6 (40.0%)	2 (28.6%)	
Type of heavy chain					0.702
	IgG	65 (58.6%)	11 (73.3%)	5 (71.4%)	
	IgA	21 (18.9%)	3 (20.0%)	1 (14.3%)	
	BJP	20 (18.0%)	0 (0.0%)	1 (14.3%)	
	IgM	1 (0.9%)	0 (0.0%)	0 (0.0%)	
	IgD	3 (2.7%)	0 (0.0%)	0 (0.0%)	
	NA	1 (0.9%)	1 (6.7%)	0 (0.0%)	
Type of light chain					0.139
	λ	41 (36.6%)	2 (13.3%)	0 (0.0%)	
	κ	68 (60.7%)	12 (80.0%)	7 (100.0%)	
	NA	3 (2.7%)	1 (6.7%)	0 (0.0%)	
ISS stage at diagnosis					0.121
	I	35 (31.3%)	3 (21.4%)	4 (57.1%)	
	II	34 (30.4%)	8 (57.1%)	0 (0.0%(	
	III	34 (30.4%)	3 (21.4%)	2 (28.6%)	
	NA	9 (8.0%)	0 (0.0%)	1 (14.3%)	
High-risk cytogenic abnormality					0.354
	none	47 (42.0%)	8 (53.3%)	3 (42.9%)	
	at least one	40 (35.7%)	6 (40.0%)	1 (14.3%)	
	NA	25 (22.3%)	1 (6.7%)	3 (42.9%)	

Laboratory data before isatuximab treatment					
White blood cell count	(/μL, median, range)	4045 (940-13230)	4300 (1300-10300)	3400 (900-5300)	0.599
Lymphocyte/monocyte ratio	(median, range)	2.36 (0.05-193.0)	3.18 (0.29-35.0)	1.95 (1.42-17.5)	0.852
Neutrophil/lymphocyte ratio	(median, range)	2.37 (0.03-67.0)	1.88 (0.36-45.0)	2.10 (0.68-8.30)	0.853
Free light chain	(mg/L, median, range)				
	κ	2.3 (0.5-12040)	193 (0.5-3680)	692 (42-1606)	0.003
	λ	9.7 (0.4-19665)	9.4 (0.5-69)	7.2 (0.7-19)	0.432
	κ/λ ratio	2.3 (0.001-14171)	49.5 (0.01-4089)	85.9 (6.0-306)	0.063
B2MG	(mg/L, median, range)	3.5 (1.3-31.8)	3.1 (1.5-12.0)	5.1 (0.2-6.3)	0.856
Prior regimen numbers					0.023
	median (range)	7 (2-18)	5 (3-18)	4 (2-7)	
Prior use of daratumumab					0.027
	Yes	69 (62.2%)	13 (86.7%)	2 (28.6%)	
Follow-up period of survivor					0.002
	median days (range)	612 (14-1137)	182 (7-600)	146 (10-518)	

The characteristics of multiple myeloma patients according to the regimen are shown in Supplementary Table 1.

NA: not available; ISS: International Staging System; β_2 microglobulin: B2MG.

Supplementary Table 2. Univariate analysis for PFS under the Isa-KD and Isa-D regimens

		Isa-KD regimen			Isa-D regimen		
Factors		**1-year-PFS (%)**	**95% CI**	**p-value**	**1-year-PFS (%)**	**95% CI**	**p-value**
Age	<65 years	60.0	12.6-88.2	0.604	66.7	5.4-94.5	0.922
	≥65 years	62.2	21.3-86.4		NA	NA	
Gender	male	62.5	22.9-86.1	0.708	50.0	5.8-84.5	0.617
	female	62.5	14.2-89.3		NA	NA	
High-risk cytogenic abnormalities	none	46.9	12.0-76.3	0.448	NA	NA	0.117
	at least one	80.0	20.4-96.9		NA	NA	
	NA	NA	NA		NA	NA	
White blood cell counts	<3000/μl	100.0	NA	0.318	NA	NA	0.695
	≥3000/μl	58.2	21.3-82.7		66.7	5.4-94.5	
Lymphocyte/monocyte ratio	≥4	75.0	12.8-96.1	0.756	NA	NA	0.617
	<4	62.2	21.3-86.4		50.0	5.8-84.5	
Neutrophil/lymphocyte ratio	<2.3	44.4	6.6-78.5	0.177	NA	NA	0.117
	≥2.3	85.7	33.4-97.9		100.0	NA	
ISS Stage	I	100.0	NA	0.113	66.7	5.4-94.5	0.515
	II	38.1	6.1-71.6		NA	NA	
	III	100.0	NA		NA	NA	
κ/λ ratio	0.1-10	100.0	NA	0.311	NA	NA	1.000
	≤0.1, ≥10	56.2	14.7-84.2		NA	NA	
B2MG	<3.5mg/L	100.0	NA	0.199	100.0	NA	0.225
	≥3.5mg/L	33.3	0.9-77.4		NA	NA	

Prior regimen numbers	<6	71.1	23.3-92.3	0.103	66.7	5.4-94.5	0.046
	≥6	40.0	5.2-75.3		NA	NA	
Prior use of daratumumab	no	50.0	0.6-91.0	0.795	37.5	1.1-80.8	0.388
	yes	62.5	26.8-84.6		NA	NA	

Progression-free survival (PFS) was calculated from the time of isatuximab treatment to the progression of the disease. Univariate analyses against PFS in MM patients treated with the Isa-KD and Isa-D regimens were performed for each factor. The log-rank test was used for comparisons among groups. One-year PFS (%) is shown with the 95% confidence interval (CI) and p-value.

PFS: progression-free survival; CI: confidence interval; ISS: International Staging System; B2MG: β2 microglobulin; NA: not available.

Supplementary Table 3. Univariate analysis for OS under the Isa-KD and Isa-D regimens

		**Isa-KD regimen**			**Isa-D regimen**		
Factors		**1-year-OS (%)**	**95% CI**	**p-value**	**1-year-OS (%)**	**95% CI**	**p-value**
Age	<65 years	100.0	NA	0.343	66.7	5.4-94.5	0.221
	≥65 years	70.0	22.5-91.8		NA	NA	
Gender	male	80.0	20.4-96.9	0.750	37.5	1.1-80.8	0.388
	female	83.3	27.3-97.5		100.0	NA	
High-risk cytogenic abnormalities	none	83.3	27.3-97.5	0.877	66.7	5.4-94.5	0.623
	at least one	66.7	5.4-94.5		NA	NA	
	NA	NA	NA		NA	NA	
White blood cell counts	<3000/μl	100.0	NA	0.516	50.0	0.6-91.0	0.695
	≥3000/μl	71.1	23.3-92.3		50.0	0.6-91.0	
Lymphocyte/monocyte ratio	≥4	66.7	5.4-94.5	0.734	NA	NA	0.388
	<4	87.5	38.7-98.1		37.5	1.1-80.8	
Neutrophil/lymphocyte ratio	<2.3	80.0	20.4-96.9	0.757	100.0	NA	0.281
	≥2.3	75.0	12.8-96.1		33.3	0.9-77.4	
ISS Stage	I	100.0	NA	0.549	50.0	0.6-91.0	0.083
	II	80.0	20.4-96.9		NA	NA	
	III	100.0	NA		NA	NA	
κ/λ ratio	0.1-10	100.0	NA	0.593	NA	NA	1.000
	≤0.1, ≥10	85.7	33.4-97.9		37.5	1.1-80.8	
B2MG	<3.5mg/L	100.0	NA	0.414	100.0	NA	0.225
	≥3.5mg/L	66.7	5.4-94.5		NA	NA	

Prior regimen numbers	<6	75.0	12.8-96.1	0.728	75.0	12.8-96.1	0.450
	≥6	75.0	12.8-96.1		NA	NA	
Prior use of daratumumab	no	50.0	0.6-91.0	0.350	50.0	5.8-84.5	0.617
	yes	90.0	47.3-98.5		NA	NA	

Overall survival (OS) was calculated from the time of isatuximab treatment to the time of death by any cause. Univariate analyses against PFS in MM patients treated with the Isa-KD and Isa-D regimens were performed for each factor. The log-rank test was used for comparisons among groups. One-year-OS (%) is shown with the 95% confidence interval (CI) and p-value.

OS: overall survival; CI: confidence interval; ISS: International Staging System; B2MG: β2 microglobulin; NA: not available.
